# 1*H*-Pyrrole-2-carbohydrazide

**DOI:** 10.1107/S1600536811002650

**Published:** 2011-01-26

**Authors:** Lina Wang, Xiangrong Liu, Chun Yang, Shunsheng Zhao, Kanshe Li

**Affiliations:** a College of Chemistry and Chemical Engineering, Xi’an University of Science & Technology, Xi’an 710054, People’s Republic of China

## Abstract

The title compound, C_5_H_7_N_3_O, was obtained by the reaction of ethyl 1*H*-pyrrol-2-carboxyl­ate and hydrazide hydrate. In the crystal, mol­ecules are linked *via* inter­molecular N—H⋯N and N—H⋯O hydrogen bonds, forming a supra­molecular grid.

## Related literature

For background to pyrrole derivatives and their biological activity, see: Joshi *et al.* (2008 ▶); Demirayak *et al.* (1999 ▶); Halazy & Magnus (1984 ▶); Bijev (2006 ▶); Sbardella *et al.* (2004 ▶).
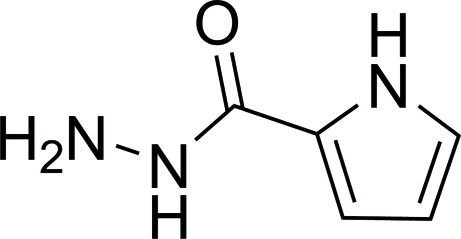


## Experimental

### 

#### Crystal data

C_5_H_7_N_3_O
*M* *_r_* = 125.14
Orthorhombic, 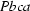

*a* = 9.9789 (16) Å
*b* = 8.5633 (14) Å
*c* = 13.657 (2) Å
*V* = 1167.0 (3) Å^3^
*Z* = 8
Mo *K*α radiation
μ = 0.11 mm^−1^
*T* = 296 K
0.31 × 0.28 × 0.16 mm

#### Data collection

Bruker APEXII CCD diffractometer
Absorption correction: multi-scan (*SADABS*; Sheldrick, 2004 ▶) *T*_min_ = 0.968, *T*_max_ = 0.983
5327 measured reflections
1043 independent reflections
758 reflections with *I* > 2σ(*I*)
*R*_int_ = 0.031

#### Refinement

*R*[*F* ^2^ > 2σ(*F* ^2^)] = 0.043
*wR*(*F* ^2^) = 0.146
*S* = 1.04
1043 reflections
90 parameters
H atoms treated by a mixture of independent and constrained refinement
Δρ_max_ = 0.16 e Å^−3^
Δρ_min_ = −0.21 e Å^−3^

### 

Data collection: *APEX2* (Bruker, 2001 ▶); cell refinement: *SAINT* (Bruker, 2001 ▶); data reduction: *SAINT*; program(s) used to solve structure: *SHELXS97* (Sheldrick, 2008 ▶); program(s) used to refine structure: *SHELXL97* (Sheldrick, 2008 ▶); molecular graphics: *SHELXTL* (Sheldrick, 2008 ▶); software used to prepare material for publication: *SHELXTL*.

## Supplementary Material

Crystal structure: contains datablocks I, global. DOI: 10.1107/S1600536811002650/fy2001sup1.cif

Structure factors: contains datablocks I. DOI: 10.1107/S1600536811002650/fy2001Isup2.hkl

Additional supplementary materials:  crystallographic information; 3D view; checkCIF report

## Figures and Tables

**Table 1:** Hydrogen-bond geometry (Å, °)

*D*—H⋯*A*	*D*—H	H⋯*A*	*D*⋯*A*	*D*—H⋯*A*
N1—H1⋯N3^i^	0.86	2.15	2.996 (2)	169
N2—H2⋯O1^ii^	0.86	2.06	2.8422 (19)	151
N3—H3*B*⋯O1^iii^	0.91 (3)	2.12 (3)	3.023 (3)	168 (2)

Symmetry codes: (i) 
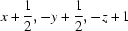
; (ii) 
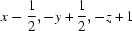
; (iii) 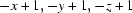.

**Fig. 1.**
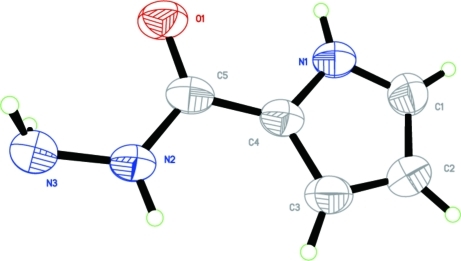
The molecular structure for 2-pyrrole hydrazide, with atom labels and 50% probability displacement ellipsoids for non-H atoms.

**Fig. 2.**
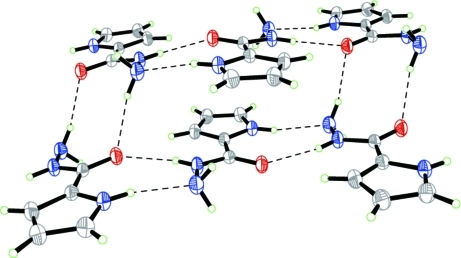
View of the hydrogen bonds for 2-pyrrole hydrazide, H atoms not involved in hydrogen bonding have been omitted.

**Fig. 3.**
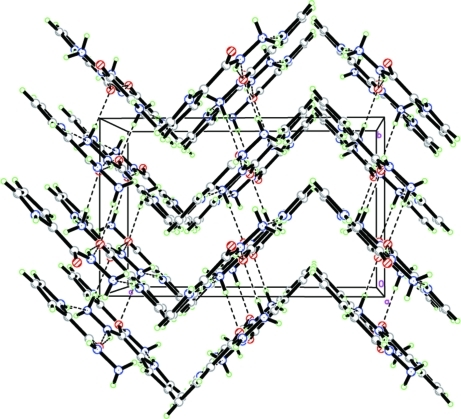
The packing for 2-pyrrole hydrazide, viewed down the a axis.

## supplementary crystallographic information

### Comment

Pyrrole is one of the most ubiquitous heterocycles in the plant and animal kingdom because of its participation as a subunit of chlorophyll in plant cells and hemin and vitamin B12 in animal cells (Joshi *et al.*, 2008). Pyrrole and its derivatives have shown to possess biological activities such as antibacterial (Demirayak *et al.*, 1999), antitumor (Halazy *et al.*, 1984), analgesics, antitubercular (Bijev, 2006), anti-inflammatory, and antiallergic (Sbardella *et al.*, 2004). Several macromolecular antibiotics having pyrrole structure were isolated from biological sources and their activities were defined.

The molecular structure for 2-pyrrole hydrazide is shown in Fig. 1. The crystal structure is stabilized by N1—H1···N3, N2—H2···O1 and N3—H3B···O1 hydrogen bonds, as shown in Fig. 2 and Table 1.

In addition, as shown in Fig.3, the packing diagram of the title compound looks like the wave viewed down the *a* axis.

### Experimental

To a 25 mL round-bottomed flask equipped with a magnetic stirrer, 0.5 mL of hydrazine hydrate (80% in water) and 0.1392 g of 1*H*-pyrrol-2-carboxlic acid ethyl ester (1 mmol) were added. Then the temperature of the mixture was elevated to 70°C for 45 min and the mixture was cooled to room temperature. The formed suspension was filtered off, washed with Et_2_O, and recrystallized from absolute ethyl alcohol. 0.113 g of the hydrazide was obtained with a yield of 90%.

### Refinement

H atoms attached to N3 were located in a difference Fourier map. All other H atoms were placed at calculated positions and all were refined in riding model, with N—H and C—H distances in the range of 0.86 and 0.93 Å and *U*_iso_(H)= 1.2 *U*_eq_ of the attached N and C atoms.

### Crystal data

**Table:**

C_5_H_7_N_3_O	*D*_x_ = 1.424 Mg m^−^^3^
*M**_r_* = 125.14	Mo *K*α radiation, λ = 0.71073 Å
Orthorhombic, *P**b**c**a*	Cell parameters from 990 reflections
*a* = 9.9789 (16) Å	θ = 3.0–22.4°
*b* = 8.5633 (14) Å	µ = 0.11 mm^−^^1^
*c* = 13.657 (2) Å	*T* = 296 K
*V* = 1167.0 (3) Å^3^	Block, colourless
*Z* = 8	0.31 × 0.28 × 0.16 mm
*F*(000) = 528	

### Data collection

**Table:**

Bruker APEXII CCD diffractometer	1043 independent reflections
Radiation source: fine-focus sealed tube	758 reflections with *I* > 2σ(*I*)
graphite	*R*_int_ = 0.031
φ and ω scans	θ_max_ = 25.1°, θ_min_ = 3.0°
Absorption correction: multi-scan (*SADABS*; Sheldrick, 2004)	*h* = −11→11
*T*_min_ = 0.968, *T*_max_ = 0.983	*k* = −6→10
5327 measured reflections	*l* = −16→16

### Refinement

**Table:**

Refinement on *F*^2^	Primary atom site location: structure-invariant direct methods
Least-squares matrix: full	Secondary atom site location: difference Fourier map
*R*[*F*^2^ > 2σ(*F*^2^)] = 0.043	Hydrogen site location: inferred from neighbouring sites
*wR*(*F*^2^) = 0.146	H atoms treated by a mixture of independent and constrained refinement
*S* = 1.04	*w* = 1/[σ^2^(*F*_o_^2^) + (0.0957*P*)^2^] where *P* = (*F*_o_^2^ + 2*F*_c_^2^)/3
1043 reflections	(Δ/σ)_max_ < 0.001
90 parameters	Δρ_max_ = 0.16 e Å^−^^3^
0 restraints	Δρ_min_ = −0.21 e Å^−^^3^

### Special details

**Table:**

Geometry. All e.s.d.'s (except the e.s.d. in the dihedral angle between two l.s. planes) are estimated using the full covariance matrix. The cell e.s.d.'s are taken into account individually in the estimation of e.s.d.'s in distances, angles and torsion angles; correlations between e.s.d.'s in cell parameters are only used when they are defined by crystal symmetry. An approximate (isotropic) treatment of cell e.s.d.'s is used for estimating e.s.d.'s involving l.s. planes.
Refinement. Refinement of *F*^2^ against ALL reflections. The weighted *R*-factor *wR* and goodness of fit *S* are based on *F*^2^, conventional *R*-factors *R* are based on *F*, with *F* set to zero for negative *F*^2^. The threshold expression of *F*^2^ > σ(*F*^2^) is used only for calculating *R*-factors(gt) *etc*. and is not relevant to the choice of reflections for refinement. *R*-factors based on *F*^2^ are statistically about twice as large as those based on *F*, and *R*- factors based on ALL data will be even larger.

### Fractional atomic coordinates and isotropic or equivalent isotropic displacement parameters (Å^2^)

**Table:**

	*x*	*y*	*z*	*U*_iso_*/*U*_eq_	
N1	0.55417 (12)	0.04787 (18)	0.64606 (11)	0.0469 (5)	
H1	0.6352	0.0752	0.6332	0.056*	
N2	0.34109 (14)	0.28224 (17)	0.48978 (11)	0.0495 (5)	
H2	0.2667	0.2414	0.5087	0.059*	
N3	0.33807 (16)	0.3955 (2)	0.41573 (15)	0.0551 (5)	
O1	0.56366 (11)	0.29286 (15)	0.50912 (10)	0.0538 (5)	
C1	0.5178 (2)	−0.0654 (2)	0.71020 (14)	0.0523 (5)	
H1A	0.5761	−0.1266	0.7470	0.063*	
C2	0.3816 (2)	−0.0744 (2)	0.71164 (14)	0.0544 (6)	
H2A	0.3304	−0.1421	0.7495	0.065*	
C3	0.33310 (17)	0.0371 (2)	0.64571 (14)	0.0510 (6)	
H3	0.2435	0.0573	0.6318	0.061*	
C4	0.44238 (15)	0.1116 (2)	0.60516 (13)	0.0418 (5)	
C5	0.45432 (16)	0.2352 (2)	0.53214 (13)	0.0423 (5)	
H3A	0.394 (3)	0.359 (3)	0.3665 (19)	0.086 (8)*	
H3B	0.380 (3)	0.483 (3)	0.4387 (19)	0.095 (9)*	

### Atomic displacement parameters (Å^2^)

**Table:**

	*U*^11^	*U*^22^	*U*^33^	*U*^12^	*U*^13^	*U*^23^
N1	0.0320 (8)	0.0562 (10)	0.0526 (10)	−0.0009 (7)	0.0010 (6)	0.0026 (8)
N2	0.0285 (8)	0.0569 (10)	0.0631 (10)	−0.0010 (6)	0.0003 (6)	0.0119 (8)
N3	0.0383 (10)	0.0595 (12)	0.0674 (12)	−0.0003 (8)	−0.0021 (8)	0.0121 (10)
O1	0.0304 (8)	0.0574 (9)	0.0737 (10)	−0.0019 (5)	0.0016 (6)	0.0054 (7)
C1	0.0483 (11)	0.0604 (12)	0.0481 (11)	0.0021 (9)	−0.0002 (8)	0.0056 (10)
C2	0.0455 (12)	0.0631 (13)	0.0545 (12)	−0.0060 (9)	0.0063 (9)	0.0041 (10)
C3	0.0357 (10)	0.0618 (12)	0.0554 (12)	−0.0037 (9)	0.0001 (8)	−0.0008 (10)
C4	0.0336 (9)	0.0469 (11)	0.0447 (10)	−0.0001 (7)	−0.0003 (7)	−0.0048 (8)
C5	0.0319 (10)	0.0452 (10)	0.0497 (11)	0.0003 (8)	0.0005 (7)	−0.0091 (9)

### Geometric parameters (Å, °)

**Table:**

N1—C1	1.356 (2)	O1—C5	1.2380 (18)
N1—C4	1.362 (2)	C1—C2	1.361 (3)
N1—H1	0.8600	C1—H1A	0.9300
N2—C5	1.332 (2)	C2—C3	1.399 (3)
N2—N3	1.402 (2)	C2—H2A	0.9300
N2—H2	0.8600	C3—C4	1.380 (2)
N3—H3A	0.93 (3)	C3—H3	0.9300
N3—H3B	0.91 (3)	C4—C5	1.459 (3)
			
C1—N1—C4	109.41 (15)	C1—C2—C3	107.33 (17)
C1—N1—H1	125.3	C1—C2—H2A	126.3
C4—N1—H1	125.3	C3—C2—H2A	126.3
C5—N2—N3	122.74 (15)	C4—C3—C2	107.48 (16)
C5—N2—H2	118.6	C4—C3—H3	126.3
N3—N2—H2	118.6	C2—C3—H3	126.3
N2—N3—H3A	106.1 (15)	N1—C4—C3	107.30 (17)
N2—N3—H3B	108.1 (17)	N1—C4—C5	120.28 (14)
H3A—N3—H3B	104 (2)	C3—C4—C5	132.42 (15)
N1—C1—C2	108.47 (17)	O1—C5—N2	121.13 (18)
N1—C1—H1A	125.8	O1—C5—C4	122.32 (15)
C2—C1—H1A	125.8	N2—C5—C4	116.54 (15)
			
C4—N1—C1—C2	−0.5 (2)	N3—N2—C5—O1	1.6 (3)
N1—C1—C2—C3	0.2 (2)	N3—N2—C5—C4	−177.42 (17)
C1—C2—C3—C4	0.2 (2)	N1—C4—C5—O1	−4.4 (3)
C1—N1—C4—C3	0.6 (2)	C3—C4—C5—O1	175.93 (18)
C1—N1—C4—C5	−179.09 (15)	N1—C4—C5—N2	174.58 (16)
C2—C3—C4—N1	−0.5 (2)	C3—C4—C5—N2	−5.1 (3)
C2—C3—C4—C5	179.19 (19)		

### Hydrogen-bond geometry (Å, °)

**Table:**

*D*—H···*A*	*D*—H	H···*A*	*D*···*A*	*D*—H···*A*
N1—H1···N3^i^	0.86	2.15	2.996 (2)	169
N2—H2···O1^ii^	0.86	2.06	2.8422 (19)	151
N3—H3B···O1^iii^	0.91 (3)	2.12 (3)	3.023 (3)	168 (2)

Symmetry codes: (i) *x*+1/2, −*y*+1/2, −*z*+1; (ii) *x*−1/2, −*y*+1/2, −*z*+1; (iii) −*x*+1, −*y*+1, −*z*+1.
